# Supplementary material for: Phylodynamics reveals extinction–recolonization dynamics underpin apparently endemic vampire bat rabies in Costa Rica
Source: Proc Biol Sci. 2019 Oct 9;286(1912):20191527. doi: 10.1098/rspb.2019.1527 (PMC6790760; doi:10.1098/rspb.2019.1527)
Supplement: Figure S1 [file rspb20191527supp6.pdf]

Streicker *et al.* Phylodynamics reveals extinction-recolonization dynamics underpin apparently endemic vampire bat rabies in Costa Rica. *Proceedings of the Royal Society B.* DOI: 10.1098/rspb.2019.1527

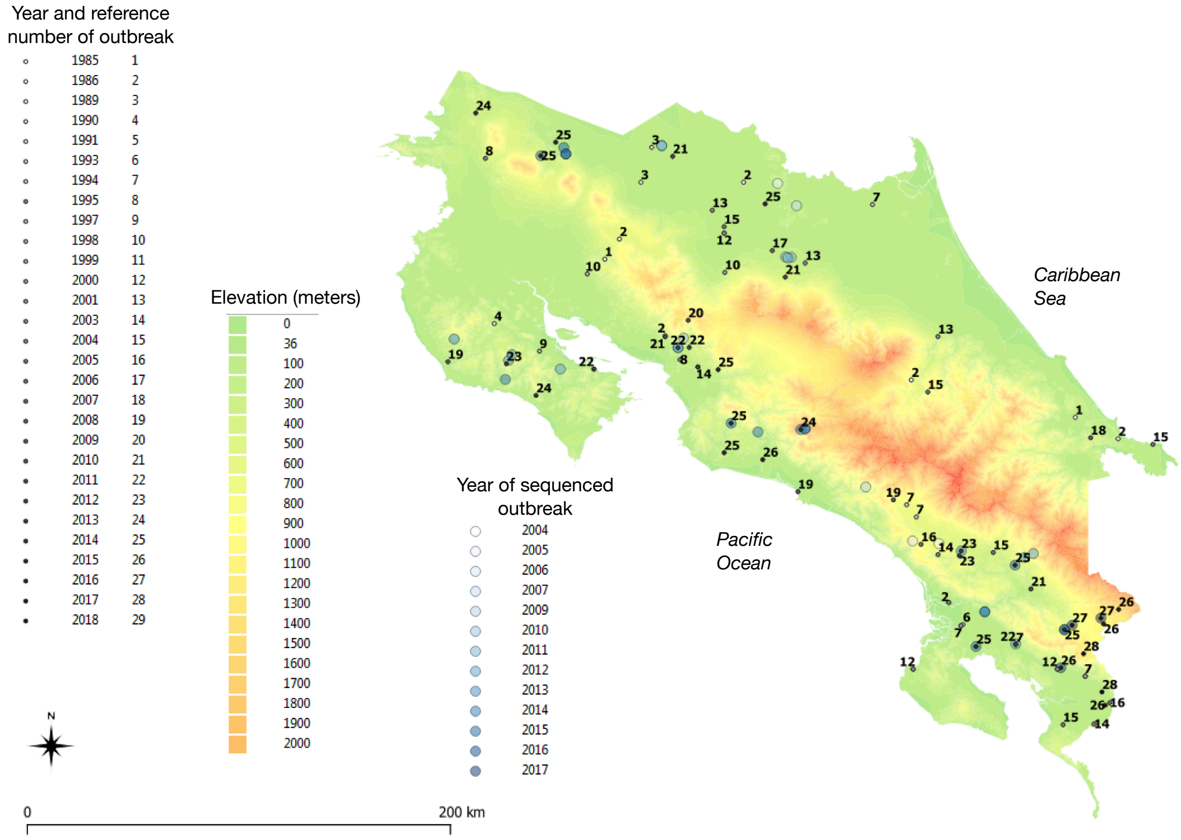

ESM Figure 1. Spatiotemporal distribution of vampire bat rabies outbreaks in Costa Rica (1985-2018). Circles and numbers indicate the year of rabies outbreaks in domestic livestock, including outbreaks for which samples were unavailable for sequencing. Outbreaks for which no sequence data were available are colored in grayscale. Outbreaks that were sequenced are colored in white to blue. In both scales, darker colors indicate more recent years. Data from the National Veterinary Service Laboratory (LANASEVE) of Costa Rica.
